# circ_0020123 promotes cell proliferation and migration in lung adenocarcinoma via PDZD8

**DOI:** 10.1515/med-2022-0434

**Published:** 2022-03-17

**Authors:** Wei Wei, Changyong Wang, Lele Wang, Jianfeng Zhang

**Affiliations:** Department of Cardiothoracic Surgery, Jinling Hospital, Nanjing University School of Medicine, Nanjing 210002, Jiangsu, China; Department of Cardiothoracic Surgery, Jinling Hospital, Nanjing University School of Medicine, 305 East Zhongshan Road, Nanjing 210002, Jiangsu, China

**Keywords:** lung adenocarcinoma, PDZD8, circ_0020123, miR-1283, ceRNA

## Abstract

High circ_0020123 expression is associated with poor prognosis in patients with non-small cell lung cancer (NSCLC) as previously reported. Whether circ_0020123 also plays an oncogenic role in lung adenocarcinoma (LUAD) is still unknown. Additionally, circ_0020123 is derived from part of exon (1312–3851) from its host gene PDZ domain-containing protein 8 (PDZD8). We hypothesized that circ_0020123 might affect malignant behaviors of LUAD cells by regulating PDZD8. Reverse transcription quantitative polymerase chain reaction revealed that PDZD8 was highly expressed in LUAD tissues and cells. PDZD8 knockdown suppressed LUAD cell proliferation and migration as shown by colony formation assays, Ethynyl deoxyuridine incorporation assays, Transwell assays, and wound healing assays. circ_0020123 was also found to be upregulated in LUAD tissues and cells. Moreover, circ_0020123 positively regulated PDZD8 expression in LUAD cells but exerted no significant effect on the transcriptional level of PDZD8. Mechanistically, circ_0020123 act as a competing endogenous RNA (ceRNA) to interact with miR-1283, thereby releasing the repression on PDZD8. Moreover, PDZD8 overexpression rescued the suppressive effect of circ_0020123 knockdown on LUAD cell proliferation and migration. In conclusion, circ_0020123 interacts with miR-1283 as a ceRNA to regulate PDZD8 expression, thus promoting the proliferation and migration of LUAD cells. The study might provide new biomarkers for future LUAD investigation.

## 1 Introduction

Lung cancer is a common malignancy with high mortality rate worldwide [1]. There were approximately 1,761,007 lung cancer-related deaths in 2018 [2]. Lung adenocarcinoma (LUAD) is a common subtype of non-small cell lung cancer (NSCLC), accounting for more than 40% of all lung cancer cases [3]. Molecular target therapy and chemotherapy for NSCLC have achieved great progress in recent years, but the 5-year survival rate of NSCLC is no more than 15% [4,5,6]. Therefore, exploring more biomarkers for NSCLC and understanding the underlying mechanisms of NSCLC pathogenesis are important for improving prognosis of NSCLC patients.

We previously found that high circ_0020123 expression is associated with poor prognosis in patients with NSCLC [7]. Herein we aimed to further explore the underlying mechanism of circ_0020123 in LUAD. Since circ_0020123 is cyclized and derived from PDZ domain-containing protein 8 (PDZD8), the functions of circ_0020123 and PDZD8 as well as their relationship in LUAD cells were investigated in the current study.

Circular RNAs (circRNAs) are single stranded RNAs with closed loop structure that have higher cellular stability than long noncoding RNAs [2]. Circular RNAs participate in the development of various cancers by regulating gene expression and interfering biological behaviors of cancer cells [8,9]. Mechanistically, circRNAs can serve as competing endogenous RNAs (ceRNAs) to release downstream messenger RNAs (mRNAs) from the repression exerted by microRNAs. In the ceRNA pattern, circRNAs regulate mRNA expression by interacting with miRNAs [10]. In recent years, many circRNAs including circ_002178 [11], circ_0013958 [12], and circ_006427 [13] have been reported to participate in the development of LUAD by serving as ceRNAs. Circ_0020123 is highly expressed in NSCLC tissues and cells according to our previous study [7]. Moreover, circ_0020123 was reported to interact with miR-144 as a ceRNA to upregulate the expression of zinc finger E-box-binding homeobox 1 (ZEB1) and enhancer of zeste 2 polycomb repressive complex 2 subunit (EZH2), thereby facilitating NSCLC progression [14]. In addition, circ_0020123 promotes NSCLC development by serving as a ceRNA of miR-488-3p to upregulate ADAM9 expression [15]. We hypothesized that circ_0020123 might function as a ceRNA to modulate its host gene PDZD8.

In the present study, we investigated the role of circ_0020123 and PDZD8 as well as their relationship in LUAD cells. This study may provide new insight into the role of circ_0020123 in LUAD diagnosis and progression.

## 2 Materials and methods

### 2.1 Bioinformatics analysis

According to circBase (http://circbase.org/), circ_0020123 is spliced from PDZD8. The genome length of circ_0020123 is 7,254 bp and the spliced length is 2,540 bp [16]. Circular RNA Interactome (CircInteractome) was employed to predict possible miRNAs binding with circ_0020123 [17]. Possible miRNAs interacting with PDZD8 were predicted with miRDB (http://mirdb.org/) [18].

### 2.2 Patients and tissue specimens

Sixteen fresh LUAD tissues and paired adjacent non-tumor lung tissues were collected from patients with LUAD at Jinling Hospital (Jiansu, China). Before operation, patients had not received any chemotherapy or radiotherapy, and the informed patient consent had been obtained. The pathological and histological diagnostics of LUAD patients were evaluated following the Revised International System for Staging Lung Cancer. The samples were preserved at −80°C before RNA extraction. The Ethics Committee in Jinling Hospital (Jiansu, China) approved the investigation.

### 2.3 Cell culture

The normal human bronchial cell line BEAS-2B and LUAD cell lines (A549 and H1975) were purchased from the American Type Culture Collection (ATCC, Manassas, VA, USA). These cells were incubated in Dulbecco’s modified Eagle’s medium (DMEM; Gibco, Gaithersburg, MD, USA) containing 10% fetal bovine serum, 100 IU/mL of 10% penicillin, and 100 µg/mL streptomycin (Invitrogen, Carlsbad, CA, USA). Cell culture was performed in a humidified atmosphere with 5% CO_2_ at 37°C.

### 2.4 Cell transfection

Short hairpin RNAs targeting PDZD8 (sh-PDZD8#1/2) or spliced form of hsa_circ_0020123 (sh-circ_0020123#1/2), miR-495 mimics, and their negative controls (sh-NC and NC mimics) were synthesized by Sangon Biotech (Shanghai, China). Empty pcDNA3.1 vectors and pcDNA3.1 vectors overexpressing PDZD8 or circ_0020123 were purchased from GenePharma (Shanghai, China). The above shRNAs (40 nM), mimics (50 nM), and vectors (10 nM) were transfected into A549 and H1975 cells using the Lipofectamine 2000 reagent (Invitrogen) according to the instructions. The transfection efficiency was examined by reverse transcription quantitative polymerase chain reaction (RT-qPCR) after 48 h.

### 2.5 RT-qPCR

TRIzol reagent (Invitrogen) was utilized to extract RNAs from A549 and H1975 cells, and a PrimeScript First Strand cDNA Synthesis Kit (Aidlab, Beijing, China) was applied to synthesize cDNA. RT-qPCR was performed using SYBR Green qPCR Mix (Invitrogen) with an ABI system (Thermo Fisher Scientific, Waltham, USA). Gene expression was calculated using the 2^−∆∆Ct^ method. GAPDH was an internal control for circ_0020123 and PDZD8, while U6 snRNA was a control set for miRNAs. Sequences of primers were presented as follows:

circ_0023123

Forward: 5′-CTTCTCCAGTTACTTGCTTGTGTAAG-3′

Reverse: 5′-GTATCTACTGTCAACCCGGCAG-3′

PDZD8

Forward: 5′-CTACCGAATTACAAGATCAGGT-3′

Reverse: 5′-ACGAAGTGTAAGTCCAACAC-3′

miR-1283

Forward: 5′-TCTACAAAGGAAAGCGCTTTCT-3′

Reverse: 5′-CTCTACAGCTATATTGCCAGCCA-3′

miR-548c-3p

Forward: 5′-CAAAAATCTCAATTACTTTTGC-3′

Reverse: 5′-CTCTACAGCTATATTGCCAGC-3′

GAPDH

Forward: 5′-TCATTTCCTGGTATGACAACGA-3′

Reverse: 5′-GGTCTTACTCCTTGGAGGC-3′

U6

Forward: 5′-GGATCAATACAGAGCAGATAAGC-3′

Reverse: 5′-CTTTCTGAATTTGCGTGCC-3′.

### 2.6 Western blot analysis

After transfection, A549 and H1975 cells were lysed with RIPA lysis buffer containing protease inhibitor. Protein concentration was determined by BCA method. Next extracted protein was subjected to 10% sodium dodecyl sulfate polyacrylamide electrophoresis gel, transferred to a polyvinylidene fluoride membrane (Bio-Rad Laboratories, Hercules, CA), and then was blocked in nonfat milk at room temperature for 2 h. Afterwards, the membrane was incubated with primary antibodies of anti-PDZD8 (PA5-46771; 1:1,000; Thermo Fisher) and anti-GAPDH (ab125247; 1:2,000; Abcam, Cambridge, MA, USA) at 4°C overnight. Subsequently, the membrane was washed and incubated with secondary antibody (Abcam) at room temperature for 2 h. The protein bands were visualized by the enhanced chemiluminescence detection system (Thermo Fisher Scientific) and analyzed by ImageJ software (National Institutes of Health, Bethesda, MA, USA). GAPDH was set as a loading control.

### 2.7 Subcellular fractionation assay

The nuclear and cytoplasmic portion of LUAD cells were extracted using a PARIS Kit (Thermo Fisher Scientific) according to instructions. Briefly, cells were incubated with lysis solution for 10 min on ice and then was centrifuged at 12,000*g* for 3 min. Cytoplasmic RNA was extracted from the supernatant and nuclear RNA was collected from nuclear pellet. The expression levels of PDZD8, GAPDH, and U6 in the cytoplasm and nuclear components were examined by RT-qPCR. GAPDH served as a cytoplasmic control, while U6 acts as a nuclear control.

### 2.8 Colony formation assay

After transfection, A549 and H1975 cells were seeded into 6-well plates (500 cells/well) and cultured in DMEM for 2 weeks. The medium was changed every 2 days. After incubation, the colonies were fixed with methanol for 30 min and stained with 3% crystal violet solution (Dingguo Biotech, Shanghai, China) for 20 min. Finally, images were taken, and the number of viable colonies (more than 50 cells per colony) was counted.

### 2.9 5-Ethynyl-2′-deoxyuridine (EdU) assay

An EdU kit (Roche, Indianapolis, IN, USA) was used to conduct the EdU assay. After indicated transfection, A549 and H1975 cells (5 × 10^4^) were incubated in DMEM with EdU solution (RiboBio) at 37°C for 4 h. Next the cells were fixed with 4% formaldehyde for 30 min and treated with glycine for 5 min. Subsequently, LUAD cells were subject to 0.5% Triton X-100 at 28°C for 10 min to make the cell membranes permeable. After being washed with phosphate buffer solution, each well was incubated with Apollo reaction cocktail for 30 min. Finally, DAPI was used to stain the nuclear DNA, and images were captured using a fluorescence microscopy (Olympus, Tokyo, Japan).

### 2.10 Transwell assay

To assess the migratory capacity of LUAD cells, 24-well Transwell chambers (BD Biosciences, Franklin Lakes, NJ, USA) were utilized. The LUAD cells (5 × 10^4^) suspended in serum-free DMEM were added to the upper chamber, and the lower chamber was supplemented with 600 µL of DMEM containing 20% fetal bovine serum. Next both the upper and lower chambers were incubated for 48 h with 5% CO_2_ and 95% humidity at 37°C. The cells on the surface of the upper chambers were carefully removed with a cotton swab. Then, the migrated cells were fixed with 10% formaldehyde and stained with 0.5% crystal violet (Beyotime) for 25 min at 24°C. The cells in 5 randomly selected views were counted under an inverted microscope (Olympus) at 200× magnification.

### 2.11 Wound healing assay

The indicated cells were cultured in 6-well plates. A sterile pipette tip was utilized to make wounds in each monolayer of cells when the cells were approximately 90% confluent. Next PBS was utilized to wash away cell debris and fresh medium was added. The distance between the two edges of the wound was measured. After 24 h, microscopic images were taken at the same field. Wound closure rate was calculated according to the formula: (the original wound areas – the actual wound areas at 24 h)/(the original wound areas).

### 2.12 Agarose gel electrophoresis

A DNeasy Blood & Tissue Kit (QIAGEN, Hilden, Germany) was utilized to extract genomic DNA (gDNA) from LUAD cells according to the instructions. TRIzol reagent (Invitrogen) was applied to extract total RNA from LUAD cells. RNA degradation and contamination were examined on 1% agarose gels.

### 2.13 RNase R treatment

The total RNA (5 µg) was incubated with or without 3 U/µg of RNase R (Epicenter Biotechnologies, WI, USA) at 37°C for 30 min. Afterwards, the RNA was purified using RNeasy MinElute Cleanup Kit (QIAGEN). At last, RNA was transcribed into cDNA, and the expression levels of PDZD8 and circ_0020123 were assessed by RT-qPCR analysis.

### 2.14 RNA immunoprecipitation (RIP) assay

An EZ-Magna RIP Kit (Millipore, Bedford, MA, USA) was utilized for the RIP assay. A549 and H1975 cells were lysed in a RIP lysis buffer. Next the cell lysate was incubated overnight at 4°C with protein G Sepharose beads precoated with Argonaute2 (Ago2) antibody. IgG antibody was set as the negative control. After the beads were washed, TRIzol reagent was utilized to extract the RNAs. At last, RT-qPCR was performed to evaluate the expression levels of the immunoprecipitated RNAs.

### 2.15 Luciferase reporter assay

The binding area between circ_0020123 and miR-1283 was predicted with CircInteractome, while that between miR-1283 and PDZD8 was predicted using miRDB. The wild-type (Wt) and mutated (Mut) fragments of miR-1283 were synthesized and, respectively, subcloned into pmirGLO vectors (Promega, Madison, WI, USA) to construct pmirGIO-miR-1283-Wt/Mut vectors. The Wt and Mut sequence of PDZD8 3′-untranslated region (UTR) containing predicted binding site with miR-1283 were subcloned into pmirGLO vectors to construct pmirGIO-PDZD8-Wt/Mut vectors. Next sh-circ_0020123#1 or sh-NC were cotransfected with miR-1283-Wt/Mut vectors into A549 and H1975 cells while PDZD8-Wt/Mut vectors were cotransfected with NC mimics, miR-1283 mimics, or miR-1283 + pcDNA3.1/circ_0020123 into LUAD cells using Lipofectamine 3000 reagent (Invitrogen) according to the manufacturer’s recommendations. The luciferase activities of miR-1283-Wt/Mut and PDZD8-Wt/Mut were assessed using a Dual Luciferase Reporter Assay Kit (Promega) after 48 h cotransfection.

### 2.16 RNA pulldown assay

Biotinylated miR-1283 WT/MUT and biotinylated negative control (bio-NC) were purchased from Sangon (Shanghai, China) and were transfected into LUAD cells. After 24 h transfection, LUAD cells were lysed as described above. Next streptavidin agarose beads (Invitrogen) were added to cell lysate for 10 min. The RNA bound to the beads were quantified by RT-qPCR.

### 2.17 Statistical analysis

Statistical analysis was performed using SPSS 19.0 software (Chicago, IL, USA). Each experiment was conducted at least three times, and data are shown as the mean value ± standard deviation. Differences between two groups were compared using Student’s *t* test, while that among more groups were analyzed by One-way analysis of variance followed by Tukey’s *post hoc* test. Spearman correlation coefficient was employed to identify gene expression correlation in LUAD tissues. The values of *p* < 0.05 were considered as statistically significant.

## 3 Results

### 3.1 PDZD8 is highly expressed in LUAD tissues and cells

**Figure 1:**
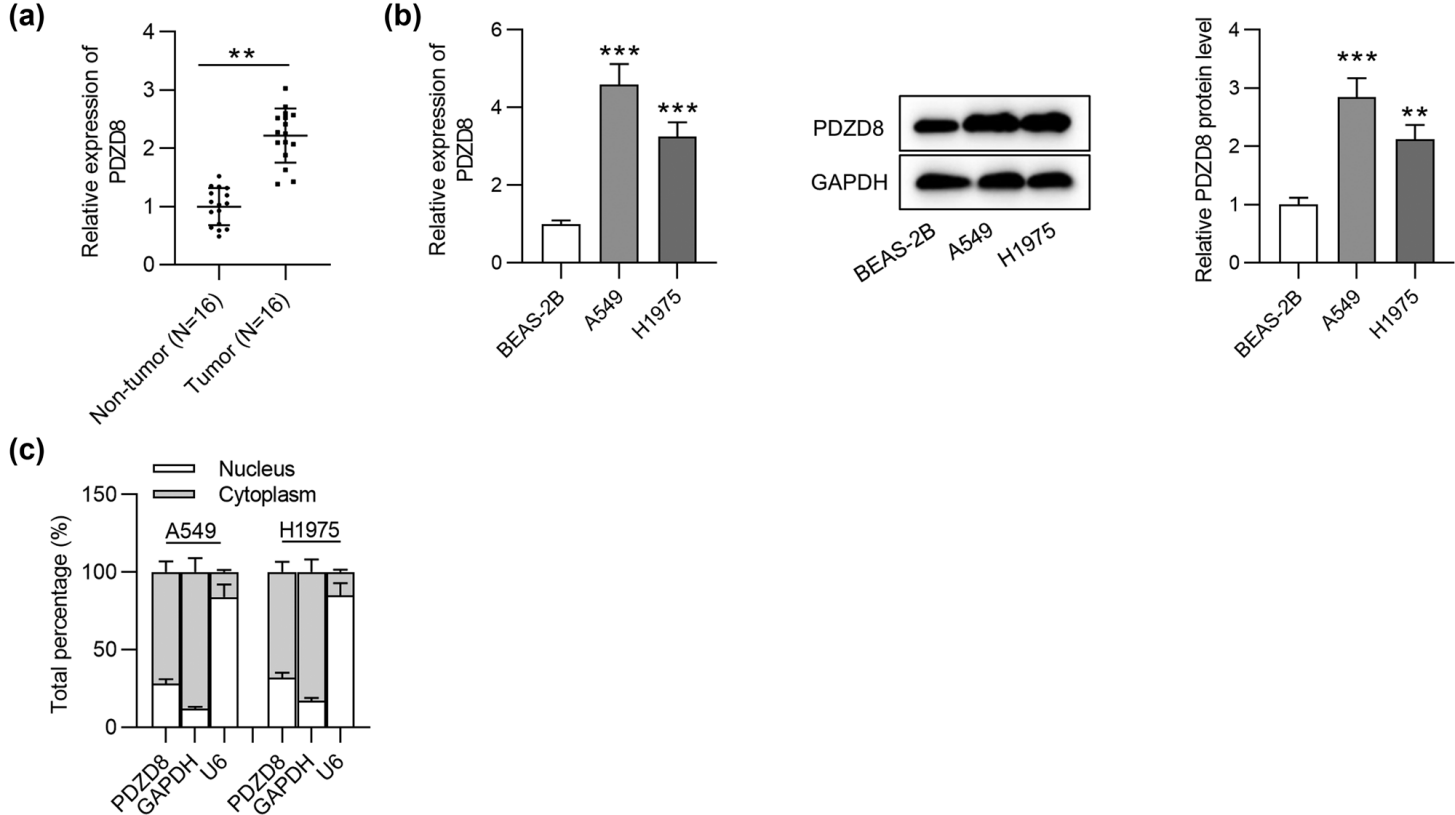
PDZD8 is highly expressed in LUAD tissues and cells. (a) PDZD8 expression in LUAD tissues (*n* = 16) was elevated compared with that in normal tissues (*n* = 16) as shown by RT-qPCR. **p < 0.01. (b) The mRNA and protein levels of PDZD8 in LUAD cells were increased as suggested by RT-qPCR and western blot analyses. **p < 0.01, ***p < 0.001 vs BEAS-2B group. (c) Subcellular fractionation assays revealed that PDZD8 was mainly distributed in cytoplasm of LUAD cells. The data are presented as the mean value ± standard deviation. Student’s *t* test was used to compare differences between LUAD tissues and normal tissues. One-way analysis of variance followed by Tukey’s *post hoc* analysis was used to compare differences among groups.

We first explored the expression of PDZD8 in LUAD tissues using RT-qPCR analysis. Compared with its expression in normal tissues, PDZD8 expression was significantly increased in LUAD tissues (Figure 1a). Next RT-qPCR and western blot analyses were performed to examine PDZD8 mRNA expression and protein levels in LUAD cells (A549 and H1975) and normal human bronchial epithelial cells (BEAS-2B). Both mRNA and protein levels of PDZD8 were much higher in LUAD cells than in normal bronchial cells (Figure 1b). Afterwards, the localization of PDZD8 in LUAD cells was explored by subcellular fractionation assays. The results revealed that PDZD8 was mainly distributed in the cytoplasm (Figure 1c).

### 3.2 PDZD8 knockdown inhibits the proliferation and migration of LUAD cells

**Figure 2:**
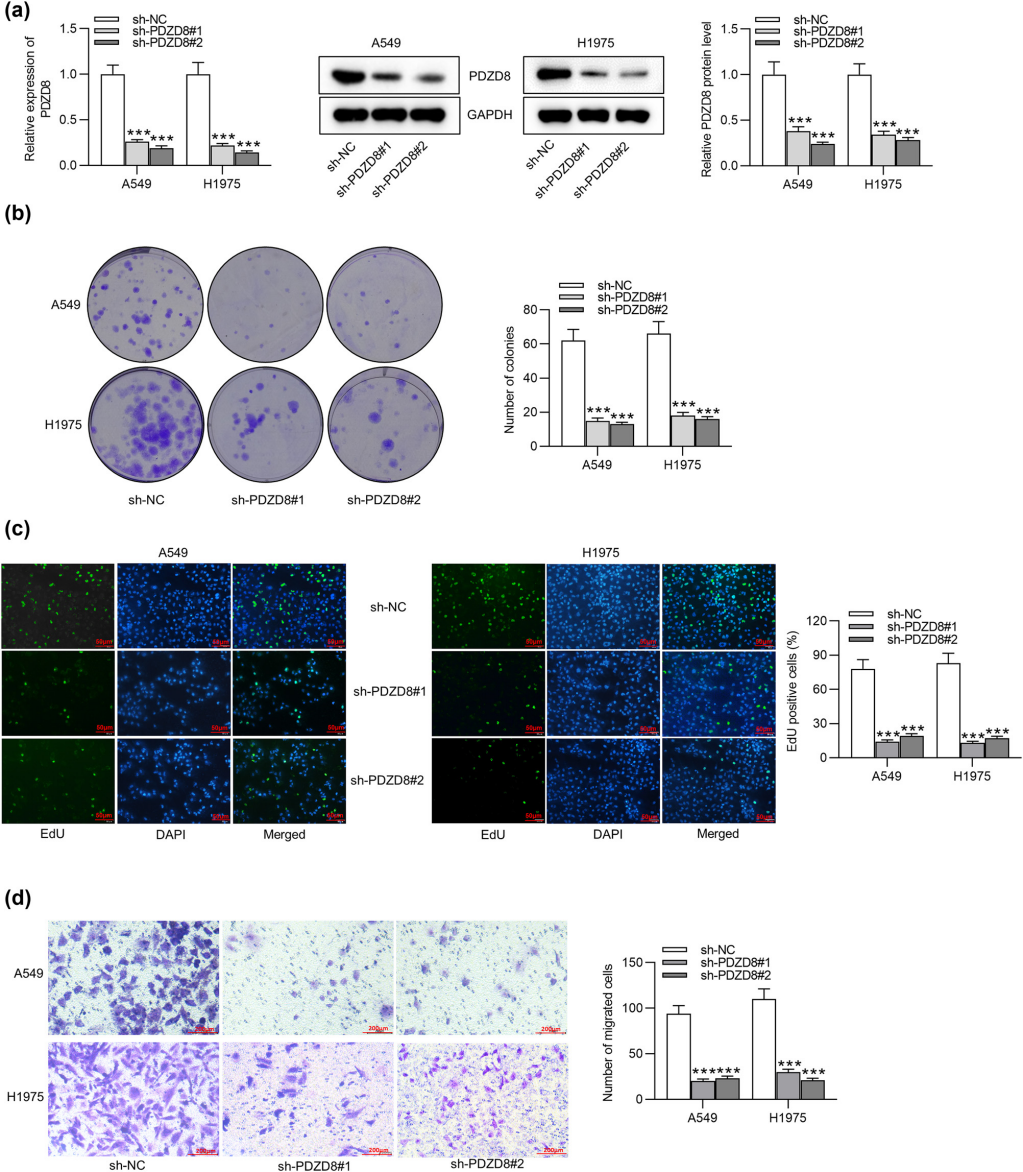

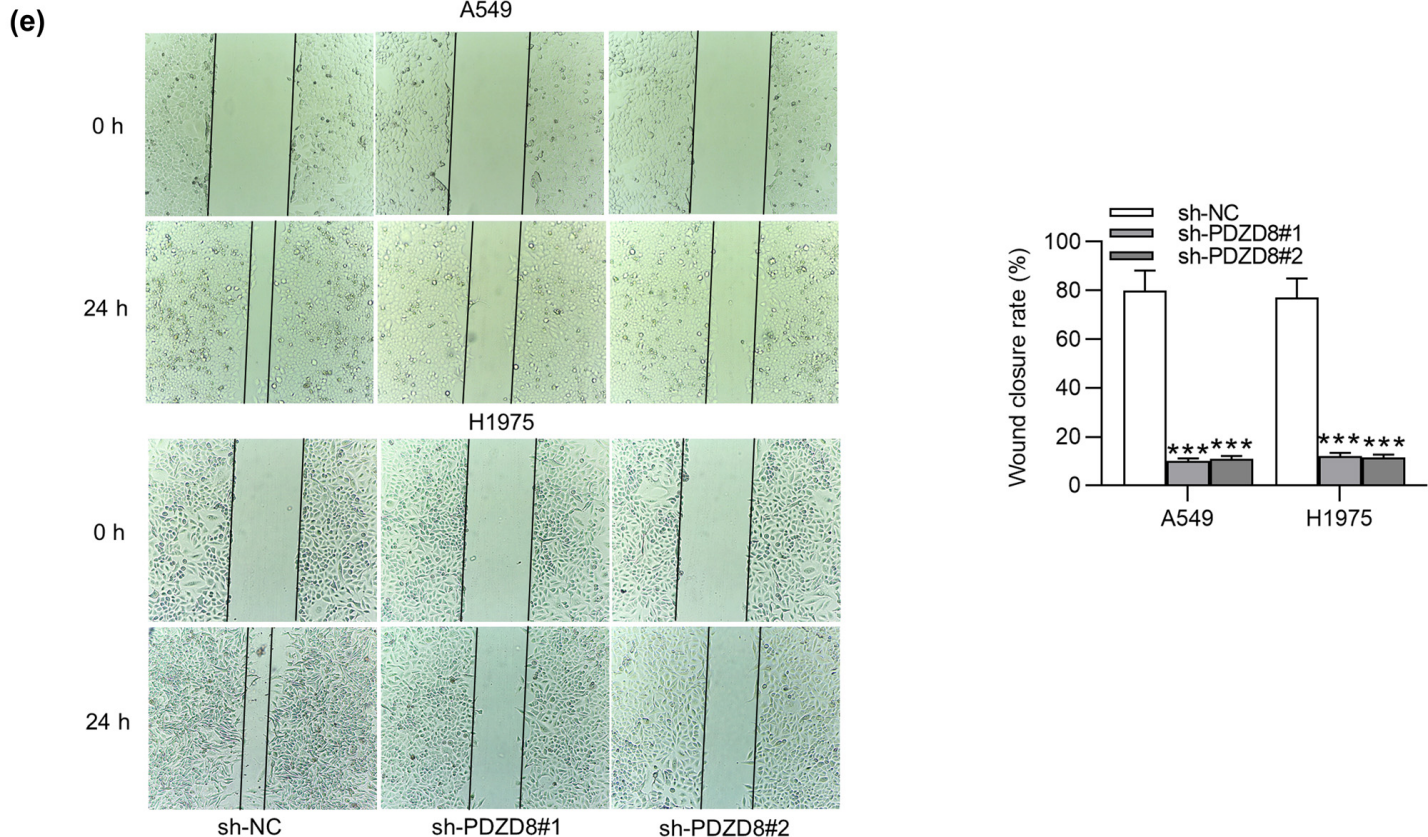
PDZD8 knockdown inhibits proliferation and migration of LUAD cells. (a) PDZD8 mRNA expression and protein levels were decreased by sh-PDZD8#1/2 in A549 and H1975 cells according to RT-qPCR and western blot. (b and c) Colony formation assays and EdU assays suggested the suppressive effect of PDZD8 knockdown on LUAD cell proliferation. (d and e) Transwell and wound healing assays revealed that PDZD8 silencing inhibited the migration of LUAD cells. The data are presented as the mean value ± standard deviation. One-way analysis of variance followed by Tukey’s *post hoc* analysis was used to compare differences among groups. ***p < 0.001 vs sh-NC group.

A series of loss-of-function assays were performed to explore the impact of PDZD8 knockdown on LUAD cell proliferation and migration. The knockdown efficiency of sh-PDZD8#1/2 was examined using RT-qPCR and western blot, and the results showed that PDZD8 was markedly decreased by sh-PDZD8#1/2 in A549 and H1975 cells (Figure 2a). Colony formation assays and EdU assays revealed that PDZD8 knockdown decreased the number of cell colonies and EdU positive cells, which indicated that PDZD8 knockdown suppressed the proliferation of LUAD cells (Figure 2b and c). As suggested by Transwell assays, the number of migrated cells was greatly reduced by PDZD8 depletion (Figure 2d). Wound healing assays showed that the wound closure rate in sh-PDZD8#1/2 group was decreased compared with that in sh-NC group (Figure 2e). The results implied that PDZD8 knockdown decreased the migratory capacity of LUAD cells.

### 3.3 circ_0020123 positively regulates PDZD8 expression in LUAD tissues and cells

**Figure 3:**
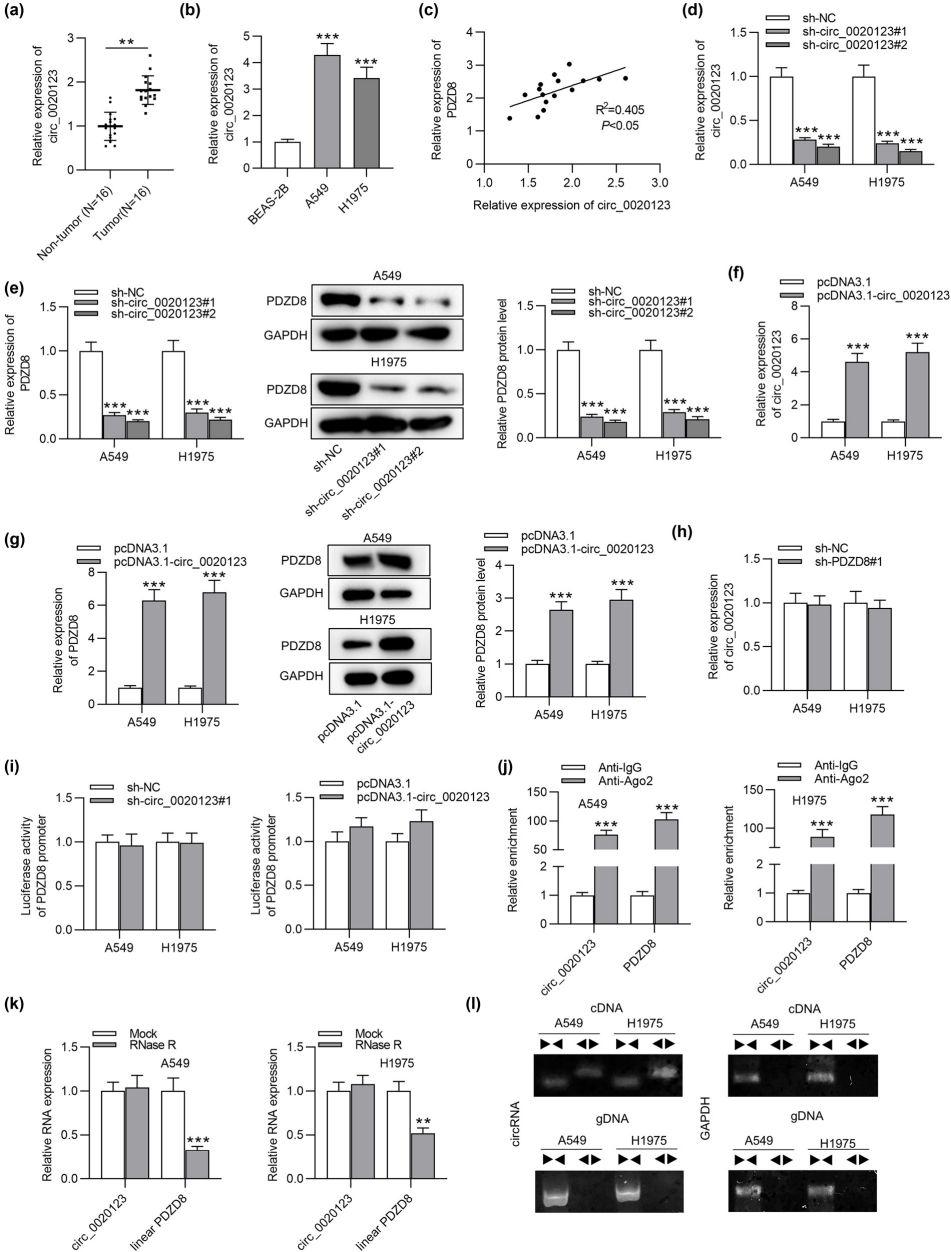
circ_0020123 positively regulates PDZD8 expression in LUAD tissues and cells. (a) circ_0020123 expression was upregulated in LUAD tissues as detected by RT-qPCR. **p < 0.01. (b) circ_0020123 showed high expression in LUAD cells as shown by RT-qPCR. ***p < 0.001 vs BEAS-2B group. (c) A positive expression correlation between circ_0020123 and PDZD8 in LUAD tissues was identified using Spearman correlation coefficient. *R* ^2^ = 0.405, *p < 0.05. (d) circ_0020123 expression was successfully knocked down in LUAD cells as shown by RT-qPCR. ***p < 0.001 vs sh-NC group. (e) circ_0020123 knockdown reduced PDZD8 expression at both the mRNA and protein levels. ***p < 0.001 vs sh-NC group. (f) RT-qPCR was performed to detect the efficiency of circ_0020123 overexpression. ***p < 0.001 vs pcDNA3.1 group. (g) The influence of overexpressed circ_0020123 on PDZD8 mRNA expression and protein levels in LUAD cells was quantified by RT-qPCR and western blot analyses. ***p < 0.001 vs pcDNA3.1 group. (h) The effect of PDZD8 knockdown on circ_0020123 expression in LUAD cells were examined by RT-qPCR. (i) Luciferase reporter assays were conducted to explore the effect of circ_0020123 on the transcription level of PDZD8. (j) RIP assays implied that circ_0020123 and PDZD8 were enriched in RNA-induced silencing complex. ***p < 0.001 vs Anti-IgG group. (k) The expression levels of circ_0020123 and linear PDZD8 in A549 and H1975 cells after RNase R treatment were determined by RT-qPCR analysis. ***p < 0.001 vs Mock group. (l) Agarose gel electrophoresis showed that circ_0020123 can be amplified by divergent primers using total cDNA, but not genomic DNA (gDNA). Pair triangles indicate the directionality of primers. The data are presented as the mean value ± standard deviation. Student’s *t* test was used to compare differences between LUAD tissues and normal tissues. One-way analysis of variance followed by Tukey’s *post hoc* analysis was used to compare differences among groups.

As suggested by RT-qPCR analysis, circ_0020123 showed higher expression in LUAD tissues than in non-tumor tissues (Figure 3a). In addition, high expression of circ_0020123 was also examined in A549 and H1975 cells compared with its expression in normal bronchial cell line BEAS-2B (Figure 3b). Subsequently, we probed the correlation between circ_0020123 and PDZD8. Based on Spearman correlation coefficient analysis, circ_0020123 expression was positively correlated with PDZD8 expression in LUAD tissues (Figure 3c). Next we knocked down circ_0020123 expression in A549 and H1975 cells via sh-circ_0020123#1/2 transfection to explore whether PDZD8 expression would be affected. The plasmid sh-circ_0020123#1/2 was designed to target the sequence around backsplice junction site. In other words, the sh-circ_0020123#1/2 targets the spliced circ_0020123 form and silences only circ_0020123. RT-qPCR revealed that circ_0020123 expression was successfully decreased with sh-circ_0020123#1/2 (Figure 3d). Western blot and RT-qPCR analyses revealed that both mRNA and protein levels of PDZD8 were downregulated by circ_0020123 knockdown in A549 and H1975 cells (Figure 3e). Next circ_0020123 expression was significantly upregulated in LUAD cells after transfection of pcDNA3.1/circ_0020123 (Figure 3f). Overexpressed circ_0020123 increased mRNA expression and protein levels of PDZD8 in LUAD cells (Figure 3g). However, silenced PDZD8 had no significant effect on circ_0020123 expression in A549 and H1975 cells (Figure 3h). Luciferase reporter assays were carried out to determine the impact of circ_0020123 on the transcription level of PDZD8. We found that the luciferase activity of PDZD8 promoter showed no response to circ_0020123 knockdown or overexpression (Figure 3i). RIP assays were conducted to further explore the relationship between circ_0020123 and PDZD8. The significant enrichment of circ_0020123 and PDZD8 in anti-Ago2 group was identified in A549 and H1975 cells, suggesting that circ_0020123 and PDZD8 were enriched in RNA-induced silencing complex (Figure 3j). Subsequently, we explored the existence of circ_0020123 in LUAD cells. After RNase R treatment, circ_0020123 was resistant to RNase R, while linear PDZD8 expression was markedly downregulated, which implied that circ_0020123 isoform was more stable than the linear PDZD8 (Figure 3k). Convergent and divergent primers were designed to amplify the linear and backsplicing forms of PDZD8 using cDNA and gDNA as the templates. The agarose gel electrophoresis showed that circ_0020123 was amplified by cDNA templates, while PDZD8 was amplified by both cDNA and gDNA templates (Figure 3l).

### 3.4 circ_0020123 acts as a ceRNA against miR-1283 to regulate PDZD8

**Figure 4:**
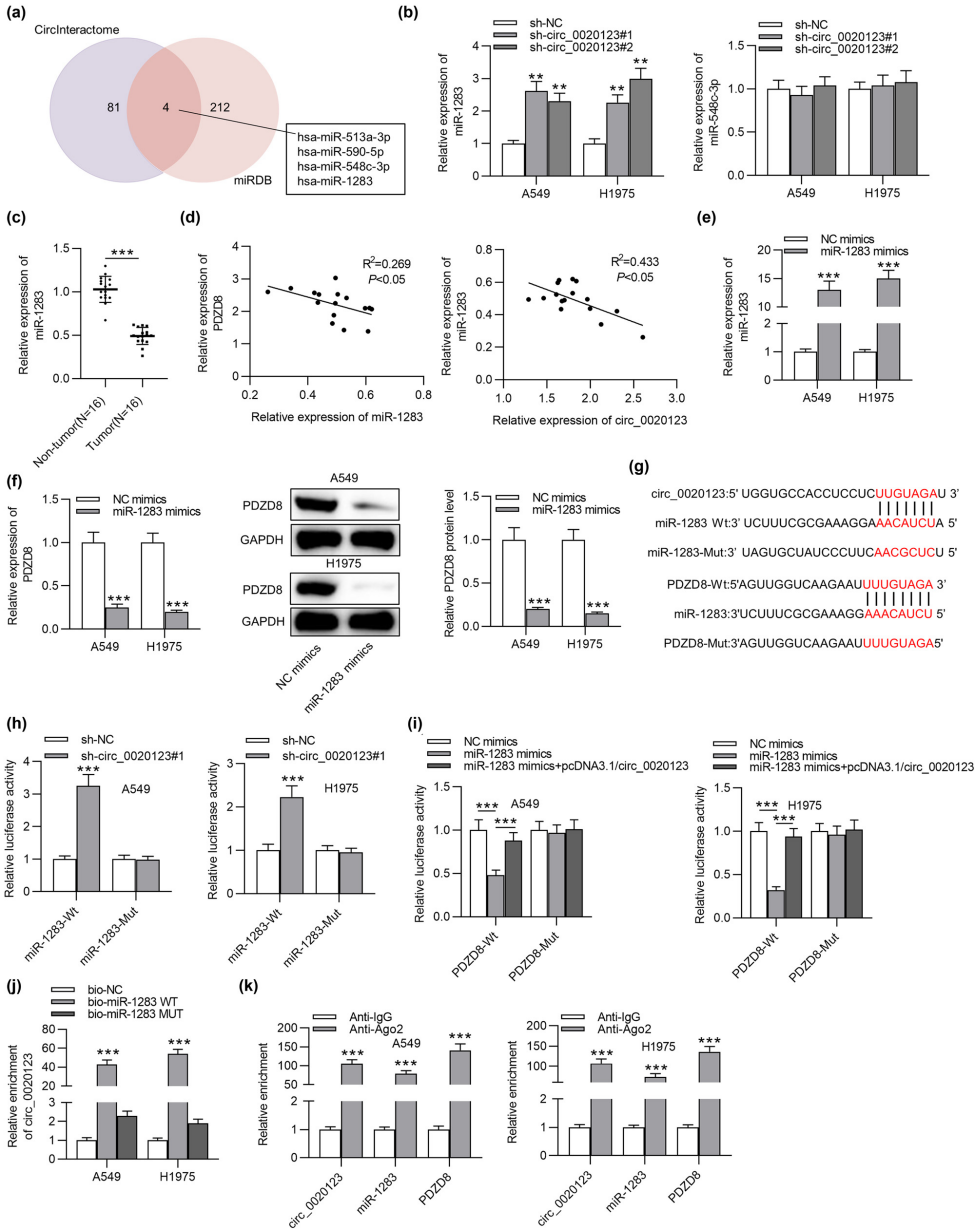
circ_0020123 acts as a ceRNA against miR-1283 to regulate PDZD8. (a) CircInteractome and miRDB databases were utilized to predict miRNAs binding with circ_0020123 and PDZD8, respectively. (b) circ_0020123 knockdown upregulated miR-1283 expression and exert no significant effect on miR-548c-3p level in LUAD cells. **p < 0.01 vs sh-NC group. (c) MiR-1283 expression in LUAD tissues was relatively low. ***p < 0.001. (d) Based on Spearman correlation coefficient, miR-1283 expression was negatively correlated with circ_0020123 expression (*R* ^2^ = 0.269, *p < 0.05) and PDZD8 expression (*R* ^2^ = 0.433, *p < 0.05) in LUAD tissues. (e) miR-1283 expression was successfully overexpressed by miR-1283 mimics in LUAD cells. ***p < 0.001 vs NC mimics group. (f) miR-1283 overexpression decreased mRNA expression and protein levels of PDZD8 in A549 and H1975 cells. ***p < 0.001 vs NC mimics group. (g) The possible binding site between miR-1283 and circ_0020123 (or PDZD8) was predicted using CircInteractome and miRDB, respectively. (h) Luciferase reporter assays indicated the binding capacities between miR-1283 and circ_0020123. ***p < 0.001 vs sh-NC group. (i) The relationship of circ_0020123, miR-1283, and PDZD8 in LUAD cells has been verified by luciferase reporter assays. ***p < 0.001 vs NC mimics group or miR-1283 mimics group. (j) The direct interaction between circ_0020123 and miR-1283 was further confirmed by RNA pulldown assays. ***p < 0.001 vs bio-NC group. (k) The enrichment of circ_0020123, miR-1283, and PDZD8 in Ago2 and IgG antibodies was probed by RIP assays. ***p < 0.001 vs Anti-IgG group. The data are presented as the mean value ± standard deviation. Student’s *t* test was used to compare differences between LUAD tissues and normal tissues. One-way analysis of variance followed by Tukey’s *post hoc* analysis was used to compare differences among groups.

To search possible miRNAs that interact with both circ_0020123 and PDZD8, CircInteractome was employed to predict miRNAs binding to circ_0020123, while miRDB was utilized to predict miRNAs interacting with PDZD8. As shown in the Venn diagram, four candidate miRNAs (miR-513a-3p, miR-590-5p, miR-548c-3p, and miR-1283) were identified (Figure 4a). In previous studies, functions of miR-513a-3p and miR-590-5p in LUAD have been investigated [19,20]. Thus, we examined miR-548c-3p and miR-1283 expression levels in A549 and H1975 cells with transfection of sh-circ_0020123#1/2. RT-qPCR suggested that miR-1283 expression was significantly increased by silencing circ_0020123, while miR-548c-3p expression was not significantly affected by circ_0020123 depletion in LUAD cells (Figure 4b). Next we detected miR-1283 expression in LUAD tissues using RT-qPCR, which suggested that miR-1283 was downregulated in LUAD tissues compared with that in normal tissues (Figure 4c). According to Spearman correlation coefficient, miR-1283 expression was negatively correlated with PDZD8 expression (or circ_0020123 expression) in LUAD tissues (Figure 4d). Next miR-1283 was overexpressed with miR-1283 mimics, and the overexpression efficiency was examined by RT-qPCR (Figure 4e). In western blot and RT-qPCR analyses, PDZD8 expression was markedly decreased at both mRNA and protein levels due to miR-1283 overexpression in LUAD cells (Figure 4f). To explore the relationship among circ_0020123, miR-1283, and PDZD8, luciferase reporter assays, RNA pulldown assays, and RIP assays were carried out. The possible binding site between circ_0020123 and miR-1283 was predicted from CircInteractome, while the binding site between miR-1283 and PDZD8 was predicted from miRDB (Figure 4g). The luciferase activity of miR-1283-Wt was significantly reduced by circ_0020123 overexpression, while no significant changes were detected in the miR-1283-Mut group in LUAD cells (Figure 4h). The luciferase activity of PDZD8-Wt was markedly downregulated due to miR-1283 overexpression and the decrease was rescued by overexpression of circ_0020123, but no significant changes happened in the activity of PDZD8-Mut (Figure 4i). The results demonstrated that circ_0020123 interacted with miR-1283 as a ceRNA to upregulate PDZD8. RNA pulldown assays were carried out to further probe the interaction between circ_0020123 and miR-1283. circ_0020123 was abundantly enriched in biotinylated miR-1283 WT group compared with its enrichment in the control Mut group (Figure 4j). The results further confirmed the direct interaction between circ_0020123 and PDZD8. Moreover, circ_0020123, miR-1283, and PDZD8 expression levels were all significantly upregulated in anti-Ago2 groups, indicating that circ_0020123, miR-1283, and PDZD8 were enriched in the RNA-induced silencing complex (Figure 4k).

### 3.5 PDZD8 overexpression rescues the inhibitory effect of circ_0020123 knockdown on LUAD cell proliferation and migration

**Figure 5:**
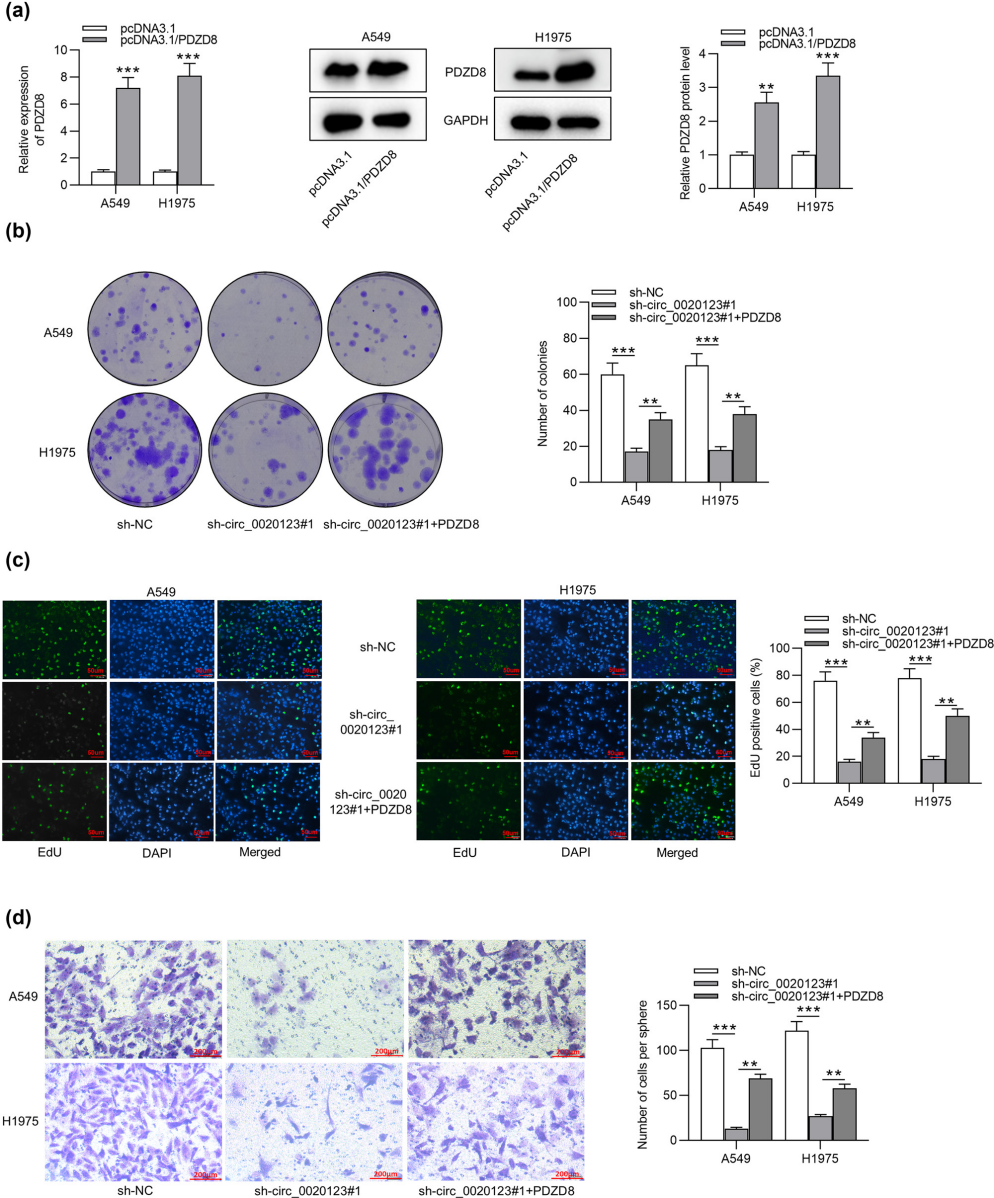

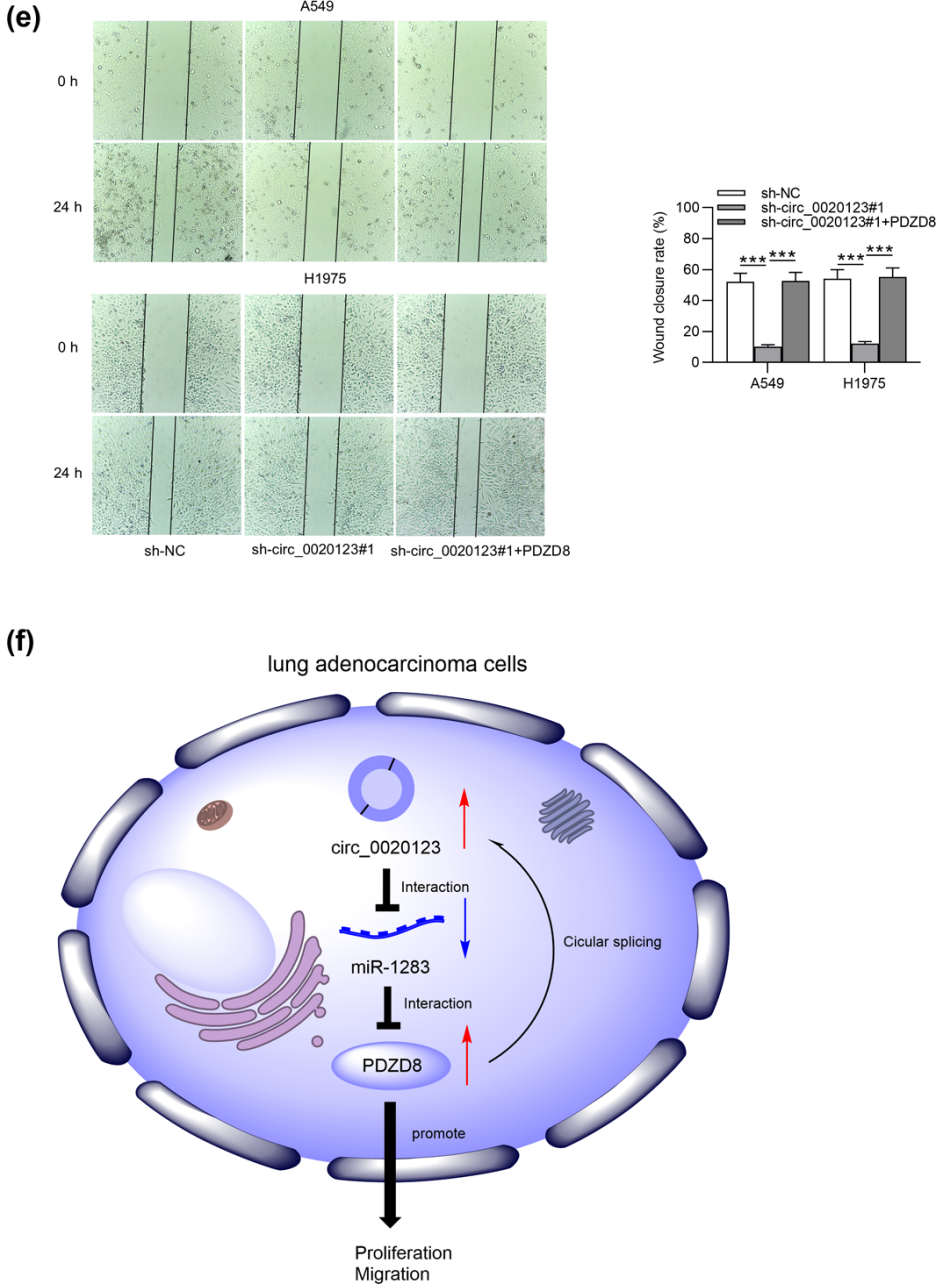
PDZD8 overexpression rescues the inhibitory effect of circ_0020123 knockdown on LUAD cell proliferation and migration. (a) The mRNA expression and protein levels of PDZD8 were increased after transfection of pcDNA3.1/PDZD8 into LUAD cells. **p < 0.01, ***p < 0.001 vs pcDNA3.1 group. (b and c) Colony formation assays and EdU assays revealed that PDZD8 overexpression rescued the inhibitory effect of circ_0020123 silencing on LUAD cell proliferation. ***p < 0.001 vs sh-NC group, **p < 0.01 vs sh-circ_0020123#1 group. (d) Transwell assays showed that the suppressive effect of circ_0020123 depletions on LUAD cell migration was reversed by PDZD8 overexpression. ***p < 0.001 vs sh-NC group, **p < 0.01 vs sh-circ_0020123#1 group. (e) Wound healing assays were performed to detect the migration of LUAD cells transfected with sh-NC, sh-circ_0020123#1, or sh-circ_0020123#1 + PDZD8. ***p < 0.001 vs sh-NC group or sh-circ_0020123#1 group. (f) The relationship of circ_0020123, miR-1283, and PDZD8 as well as their roles in LUAD cells has been illustrated by a schematic diagram. The data are presented as the mean value ± standard deviation. One-way analysis of variance followed by Tukey’s *post hoc* analysis was used to compare differences among groups.

Rescue assays were performed to determine whether circ_0020123 promotes cellular behaviors by regulating its host gene PDZD8. As shown by RT-qPCR and western blot, PDZD8 mRNA and protein levels were significantly increased after transfection of pcDNA3.1/PDZD8 (Figure 5a). Colony formation and EdU assays showed that silencing circ_0020123 inhibited LUAD cell proliferation and PDZD8 overexpression rescued the suppressive effect on cell proliferation induced by circ_0020123 knockdown (Figure 5b and c). According to Transwell and wound healing assays, circ_0020123 depletion suppressed the migration of LUAD cells and PDZD8 overexpression reversed the decrease in migratory ability induced by circ_0020123 silencing (Figure 5d and e). As shown by Figure 5f, circ_0020123 is spliced from PDZD8 and is highly expressed in LUAD cells. circ_0020123 directly interacts with miR-1283 to indirectly upregulate PDZD8. MiR-1283 is downregulated in LUAD cells and directly bind to PDZD8. In summary, circ_0020123 competes with PDZD8 for the chance of binding with miR-1283, thereby releasing PDZD8 from degradation and thus promoting cell proliferation and migration.

## 4 Discussion

LUAD is a common malignancy worldwide and its therapeutic effect still needs to be improved due to the lack of effective diagnostic and therapeutic methods [11]. CircRNA is a kind of noncoding RNA with neither a free 3′ nor 5′ end [11,21]. CircRNAs play a vital role in the progression of various cancers, including LUAD [22,23]. The current study investigated the role of circ_0020123 and its host gene PDZD8 in LUAD. PDZD8 showed high expression levels in LUAD tissues and cells and was mainly distributed in cytoplasm. PDZD8 knockdown suppressed the proliferation and migration of LUAD cells. In addition, PDZD8 expression was found to be positively correlated with circ_0020123 expression in LUAD tissues and cells. Silencing circ_0020123 downregulated both mRNA and protein levels of PDZD8 expression, while overexpressing circ_0020123 exerted the opposite effect on PDZD8 expression. Importantly, knockdown or overexpression of circ_0020123 had no effect on the transcriptional level of PDZD8. The results indicated that circ_0020123 might function as a ceRNA to regulate PDZD8.

Subsequently, we explored the shared miRNA that circ_0020123 and PDZD8 compete for in LUAD. MiRNAs are small non-protein coding RNAs that play a critical role in cancer development [24,25]. Specifically, miRNAs can promote gene degradation by binding with 3′-UTR of mRNAs at the post transcriptional level. In the ceRNA pattern, miRNAs are sponged by circRNAs, and then the suppressive effect of miRNAs on mRNAs were reversed due to circRNA-miRNA interaction [26]. Based on bioinformatics analysis, four candidate miRNAs (miR-513a-3p, miR-590-5p, miR-548c-3p, and miR-1283) that have binding site with circ_0020123 and PDZD8 were selected for this study. Among these candidate miRNAs, miR-513a-3p was reported to sensitize LUAD cells to chemotherapy by targeting GSTP1 [19], and miR-590-5p was reported to inhibit the proliferation and invasion of NSCLS cells by downregulating GAB1 expression [27]. We identified that miR-1283 was downregulated in LUAD tissues and cells. miR-1283 expression was negatively correlated with circ_0020123 expression (or PDZD8 expression) in LUAD tissues. circ_0020123 silencing upregulated miR-1283 expression in LUAD cells. MiR-1283 overexpression downregulated PDZD8 expression at both mRNA and protein levels. Moreover, circ_0020123 served as a ceRNA to bind with miR-1283, thus promoting the expression levels of PDZD8. Similar to our study, circTTBK2 promotes the development of glioma by interacting with miR-1283 and thus increases chromodomain helicase DNA binding protein 1 (CHD1) expression [28]. In addition, circGprc5a contributes to hepatocellular carcinoma progression by binding with miR-1283 to activate the YAP1/TEAD1 signaling pathway [29]. Rescue assays revealed that PDZD8 overexpression rescued the inhibitory effect of circ_0020123 silencing on LUAD cell proliferation and migration. The results suggested that circ_0020123 promotes cellular behaviors in LUAD by upregulating PDZD8. In conclusion, circ_0020123 promotes the proliferation and migration of LUAD cells by serving as a ceRNA of miR-1283 to upregulate PDZD8 expression. The study reveals the promising role of circ_0020123/miR-1283/PDZD8 axis in LUAD, which may provide potential biomarkers and therapeutic approach for LUAD.

There are some limitations in the study. First, only experiments at cellular level were conducted and *in vivo* experiments were not designed. Xenograft tumor model in nude mice will be established in future studies to explore the influence of circ_0020123/miR-1283/PDZD8 on tumor growth and metastasis *in vivo*. Second, potential downstream signaling mediated by PDZD8 was not investigated. More genes involved in the circ_0020123/miR-1283/PDZD8 axis will be further explored.

## References

[1] Xu K, Zhang C, Du T, Gabriel ANA, Wang X, Li X, et al. Progress of exosomes in the diagnosis and treatment of lung cancer. Biomed Pharmacother. 2021;134:111111. 10.1016/j.biopha.2020.111111 33352449

[2] Bray F, Ferlay J, Soerjomataram I, Siegel RL, Torre LA, Jemal A. Global cancer statistics 2018: GLOBOCAN estimates of incidence and mortality worldwide for 36 cancers in 185 countries. CA Cancer J Clin. 2018;68(6):394–424. 10.3322/caac.21492 30207593

[3] Zappa C, Mousa SA. Non-small cell lung cancer: current treatment and future advances. Transl Lung Cancer Res. 2016;5(3):288–300. 10.21037/tlcr.2016.06.07 PMC4931124 27413711

[4] Jonna S, Subramaniam DS. Molecular diagnostics and targeted therapies in non-small cell lung cancer (NSCLC): an update. Discov Med. 2019;27(148):167–70. 31095926

[5] Nagasaka M, Gadgeel SM. Role of chemotherapy and targeted therapy in early-stage non-small cell lung cancer. Expert Rev Anticancer Ther. 2018;18(1):63–70. 10.1080/14737140.2018.1409624 PMC6863145 29168933

[6] Deng X, Xiong W, Jiang X, Zhang S, Li Z, Zhou Y, et al. LncRNA LINC00472 regulates cell stiffness and inhibits the migration and invasion of lung adenocarcinoma by binding to YBX1. Cell Death Dis. 2020;11(11):945. 10.1038/s41419-020-03147-9 PMC7609609 33144579

[7] Bi R, Wei W, Lu Y, Hu F, Yang X, Zhong Y, et al. High hsa_circ_0020123 expression indicates poor progression to non-small cell lung cancer by regulating the miR-495/HOXC9 axis. Aging (Albany NY). 2020;12(17):17343–52. 10.18632/aging.103722 PMC7521531 32927434

[8] Zhao ZJ, Shen J. Circular RNA participates in the carcinogenesis and the malignant behavior of cancer. RNA biology. 2017;14(5):514–21. 10.1080/15476286.2015.1122162 PMC5449088 26649774

[9] Liu J, Li D, Luo H, Zhu X. Circular RNAs: the star molecules in cancer. Mol Aspects Med. 2019;70:141–52. 10.1016/j.mam.2019.10.006 31676107

[10] Qi X, Zhang DH, Wu N, Xiao JH, Wang X, Ma W. ceRNA in cancer: possible functions and clinical implications. J Med Gene. 2015;52(10):710–8. 10.1136/jmedgenet-2015-103334 26358722

[11] Wang J, Zhao X, Wang Y, Ren F, Sun D, Yan Y, et al. circRNA-002178 act as a ceRNA to promote PDL1/PD1 expression in lung adenocarcinoma. Cell Death Dis. 2020;11(1):32. 10.1038/s41419-020-2230-9 PMC6965119 31949130

[12] Zhu X, Wang X, Wei S, Chen Y, Chen Y, Fan X, et al. hsa_circ_0013958: a circular RNA and potential novel biomarker for lung adenocarcinoma. The FEBS journal. 2017;284(14):2170–82. 10.1111/febs.14132 28685964

[13] Yao Y, Hua Q, Zhou Y. CircRNA has_circ_0006427 suppresses the progression of lung adenocarcinoma by regulating miR-6783-3p/DKK1 axis and inactivating Wnt/β-catenin signaling pathway. Biochem Biophys Res Commun. 2019;508(1):37–45. 10.1016/j.bbrc.2018.11.079 30470570

[14] Qu D, Yan B, Xin R, Ma T. A novel circular RNA hsa_circ_0020123 exerts oncogenic properties through suppression of miR-144 in non-small cell lung cancer. Am J Cancer Res. 2018;8(8):1387–402. PMC6129481 30210911

[15] Wan J, Hao L, Zheng X, Li Z. Circular RNA circ_0020123 promotes non-small cell lung cancer progression by acting as a ceRNA for miR-488-3p to regulate ADAM9 expression. Biochem Biophys Res Commun. 2019;515(2):303–9. 10.1016/j.bbrc.2019.05.158 31153639

[16] Glažar P, Papavasileiou P, Rajewsky N. circBase: a database for circular RNAs. Rna. 2014;20(11):1666–70. 10.1261/rna.043687.113 PMC4201819 25234927

[17] Dudekula DB, Panda AC, Grammatikakis I, De S, Abdelmohsen K, Gorospe M. CircInteractome: A web tool for exploring circular RNAs and their interacting proteins and microRNAs. RNA Biol. 2016;13(1):34–42. 10.1080/15476286.2015.1128065 PMC4829301 26669964

[18] Chen Y, Wang X. miRDB: an online database for prediction of functional microRNA targets. Nucleic Acids Res. 2020;48(D1):D127–31. 10.1093/nar/gkz757 PMC6943051 31504780

[19] Zhang X, Zhu J, Xing R, Tie Y, Fu H, Zheng X, et al. miR-513a-3p sensitizes human lung adenocarcinoma cells to chemotherapy by targeting GSTP1. Lung cancer (Amsterdam, Netherlands). 2012;77(3):488–94. 10.1016/j.lungcan.2012.05.107 22749944

[20] Wang J, Zha J, Wang X. Knockdown of lncRNA NUTM2A-AS1 inhibits lung adenocarcinoma cell viability by regulating the miR-590-5p/METTL3 axis. Oncol Lett. 2021;22(5):798. 10.3892/ol.2021.13059 PMC8477074 34630705

[21] Li X, Yang L, Chen LL. The biogenesis, functions, and challenges of circular RNAs. Mol Cell. 2018;71(3):428–42. 10.1016/j.molcel.2018.06.034 30057200

[22] Liang Y, Wang H, Chen B, Mao Q, Xia W, Zhang T, et al. circDCUN1D4 suppresses tumor metastasis and glycolysis in lung adenocarcinoma by stabilizing TXNIP expression. Mol Ther Nucleic Acids. 2021;23:355–68. 10.1016/j.omtn.2020.11.012 PMC7779544 33425493

[23] Zhang SJ, Ma J, Wu JC, Hao ZZ, Zhang YN, Zhang YJ. CircRNA EPB41L2 inhibits tumorigenicity of lung adenocarcinoma through regulating CDH4 by miR-211-5p. Eur Rev Med Pharmacol Sci. 2021;25(8):3150. 10.26355/eurrev_202104_25716 33928597

[24] Tutar Y. miRNA and cancer; computational and experimental approaches. Curr Pharm Biotechnol. 2014;15(5):429. 10.2174/138920101505140828161335 25189575

[25] Lee YS, Dutta A. MicroRNAs in cancer. Annu Rev Pathol. 2009;4:199–227. 10.1146/annurev.pathol.4.110807.092222 PMC2769253 18817506

[26] Wu M, Wang G, Tian W, Deng Y, Xu Y. MiRNA-based therapeutics for lung cancer. Curr Pharm Des. 2018;23(39):5989–96. 10.2174/1381612823666170714151715 28714413

[27] Xu BB, Gu ZF, Ma M, Wang JY, Wang HN. MicroRNA-590-5p suppresses the proliferation and invasion of non-small cell lung cancer by regulating GAB1. Eur Rev Med Pharmacol Sci. 2018;22(18):5954–63. 10.26355/eurrev_201809_15926 30280777

[28] Han C, Wang S, Wang H, Zhang J. Knockdown of circ-TTBK2 inhibits glioma progression by regulating miR-1283 and CHD1. Cancer Manag Res. 2020;12:10055–65. 10.2147/CMAR.S252916 PMC7568596 33116862

[29] Lin Y, Huang G, Jin H, Jian Z. Circular RNA Gprc5a promotes HCC progression by activating YAP1/TEAD1 signalling pathway by sponging miR-1283. Onco Targets Ther. 2020;13:4509–21. 10.2147/OTT.S240261 PMC7247601 32547082

